# Current state of the evidence on community treatments for people with complex emotional needs: a scoping review

**DOI:** 10.1186/s12888-022-04171-z

**Published:** 2022-09-05

**Authors:** Sarah Ledden, Luke Sheridan Rains, Merle Schlief, Phoebe Barnett, Brian Chi Fung Ching, Brendan Hallam, Mia Maria Günak, Thomas Steare, Jennie Parker, Sarah Labovitch, Sian Oram, Steve Pilling, Sonia Johnson, the C. E. N. Mental Health Policy Research Unit Group, Alexandra Papamichail, Ava Mason, Avithaa Thayaparan, Baihan Wang, Christian Dalton Locke, Jasmine Harju-Seppänen, Jiping Mo, Magdalena Tomaskova, Natasha Lyons, Spyros Spyridonidis, Tiffeny James, Zainab Dedat, Zoë Haime

**Affiliations:** 1 Division of Psychiatry, University College London, London, UK; 2 NIHR Mental Health Policy Research Unit, Division of Psychiatry, University College London, London, UK; 3 Centre for Outcomes Research and Effectiveness, Research Department of Clinical, Educational and Health Psychology, University College London, London, UK; 4 Research Department of Primary Care & Population Health, University College London, London, UK; 5 Department of Psychology, Ludwig-Maximilians-Universität München, Munich, Germany; 6 NIHR Mental Health Policy Research Unit Co-Production Group, University College London, London, UK; 7 West London NHS Trust, London, UK; 8 NIHR Mental Health Policy Research Unit, Department of Health Service and Population Research, King’s College London, London, UK; 9 National Collaborating Centre for Mental Health, Royal College of Psychiatrists, London, UK; 10 Camden and Islington NHS Foundation Trust, London, UK

**Keywords:** Complex emotional needs, Personality disorder, Scoping review

## Abstract

**Background:**

Improving the quality of care in community settings for people with ‘Complex Emotional Needs’ (CEN—our preferred working term for services for people with a “personality disorder” diagnosis or comparable needs) is recognised internationally as a priority. Plans to improve care should be rooted as far as possible in evidence. We aimed to take stock of the current state of such evidence, and identify significant gaps through a scoping review of published investigations of outcomes of community-based psychosocial interventions designed for CEN.

**Methods:**

We conducted a scoping review with systematic searches. We searched six bibliographic databases, including forward and backward citation searching, and reference searching of relevant systematic reviews. We included studies using quantitative methods to test for effects on any clinical, social, and functioning outcomes from community-based interventions for people with CEN. The final search was conducted in November 2020.

**Results:**

We included 226 papers in all (210 studies). Little relevant literature was published before 2000. Since then, publications per year and sample sizes have gradually increased, but most studies are relatively small, including many pilot or uncontrolled studies. Most studies focus on symptom and self-harm outcomes of various forms of specialist psychotherapy: most result in outcomes better than from inactive controls and similar to other specialist psychotherapies. We found large evidence gaps. Adaptation and testing of therapies for significant groups (e.g. people with comorbid psychosis, bipolar disorder, post-traumatic stress disorder, or substance misuse; older and younger groups; parents) have for the most part only reached a feasibility testing stage. We found little evidence regarding interventions to improve social aspects of people’s lives, peer support, or ways of designing effective services.

**Conclusions:**

Compared with other longer term mental health problems that significantly impair functioning, the evidence base on how to provide high quality care for people with CEN is very limited. There is good evidence that people with CEN can be helped when specialist therapies are available and when they are able to engage with them. However, a much more methodologically robust and substantial literature addressing a much wider range of research questions is urgently needed to optimise treatment and support across this group.

**Supplementary Information:**

The online version contains supplementary material available at 10.1186/s12888-022-04171-z.

## Introduction

People who have received a diagnosis of “personality disorder” are reported to experience a range of difficulties with social functioning, mental and physical health [1, 2]. Substantial economic burdens are associated, especially due to treatment costs and productivity losses [3, 4]. Historically a “personality disorder” diagnosis was seen as indicating a lack of treatability [5]. More recently, there has been greater recognition of the needs for support and the provision of effective treatment for this group, and improving care has been identified as a priority in a variety of countries [6–9].

A heavy burden of stigma is associated with a “personality disorder” diagnosis, with negative views and discriminatory behaviour from some health professionals having especially immediate impacts [10–14]. We are sympathetic to the critique that the therapeutic nihilism and stigma accompanying a “personality disorder” diagnosis, and the lack of progress in delivering care that consistently helps rather than harms, are such that this diagnostic label—also criticised on grounds of validity—is now best left behind. Further work is needed on assessing and describing the difficulties that people who may receive this diagnostic label experience in more useful and acceptable ways: pending this, we prefer the term complex emotional needs (CEN) as a working description of the difficulties experienced by people who may receive a “personality disorder” diagnosis, and therefore use it as our headline description in this paper, as in our other publications on this topic. We are guided especially by members of our research team who have relevant lived experience in making this choice. However, the literature we have reviewed for the most part is based on “personality disorder” diagnoses of various types: thus, below we use this term where it is used in the papers included in our review.

Mental health services and mental health research are widely acknowledged not to have achieved parity in terms of resources and status with physical health care, and services for people with a “personality disorder” diagnosis are doubly disadvantaged as they appear to significantly lag behind services for people with other long-term mental health conditions [6, 15–17]. Recurrently reported difficulties include large variations in accessibility and quality of services, difficulty accessing specialist “personality disorder” services, and lack of therapeutic interventions outside them, a tendency for interventions to focus narrowly on self-harm rather than on the broader range of psychological and social outcomes that service users and carers identify as important, lack of focus on trauma experiences despite these being very frequent, and exclusion from care of people with common comorbidities such as substance misuse or bipolar disorder, or at the younger or older end of the age range [10, 17–20].

Internationally, service user activists, professional bodies and policy makers have advocated for better quality services for people with CEN [15–17]. Ideally, service improvement should be rooted in evidence-based practice [21, 22]. A number of systematic reviews have reported on the trial literature on psychological interventions for people with a “borderline personality disorder” (“BPD”) diagnosis, including Dialectal Behaviour Therapy (DBT), Mentalisation Based Therapy (MBT), Cognitive Behavioural Therapy (CBT), and psychodynamic therapies, amongst others [23–25]. Reviews tend to conclude that these specialist treatments are all more effective than treatment as usual (TAU) in achieving clinical improvements in self-harm and “borderline symptoms”, although no single intervention type has emerged as dominant [26].

However, these relatively narrowly focused systematic reviews have left unanswered a range of questions that are key to improving care holistically for the full spectrum of people who have received a “personality disorder” diagnosis, or have comparable needs [26]. Questions not addressed include how to improve important social outcomes including employment, social inclusion, relationships and parenting, and how to provide care that takes account of very frequent and extensive trauma histories. These previous reviews have also not focused on the needs of important groups, such as older adults and younger people, people with comorbidities such as substance misuse, psychosis or bipolar disorder, and people who may have received “personality disorder” diagnoses other than borderline or emotionally unstable, or who have received multiple diagnoses. The key question of service design, and what kinds of teams and networks of services most effectively meet needs and deliver continuity of care also remains largely unanswered.

Given these crucial gaps in the evidence to underpin improvement of care, our intention in the current scoping paper was to cast the net widely, seeking any quantitative evidence that may have potential as building blocks for future intervention and service design and research in this area. Our aim was to conduct a scoping review of the evidence on the effectiveness of community-based psychological interventions designed for people with CEN. In order to capture a broad range of relevant evidence, we aimed to include in our searches a broad range of diagnoses and related difficulties, interventions focused not only on self-harm and symptoms but also on social targets, and delivered at team and catchment area as well as individual levels. Observational studies can yield helpful evidence on treatment outcomes in naturalistic settings, sometimes providing pointers to interventions worth researching through randomised trials or allowing questions to be addressed, such as about area-level service design, that are difficult to investigate through trials [27]: we thus aimed also to capture evidence from such designs. We further aimed to identify preliminary investigations of feasibility and reports on adaptations of interventions to new populations or new settings, as these have potential to inform further research and intervention development. Thus, by considering this broader evidence base, we aim to take stock of what is known so far, highlight important gaps, and inform future research in this area.

## Methods

### Study design

We conducted a scoping review [28, 29] to map the evidence from studies using a range of quantitative designs on community-based treatments for CEN and to identify gaps in the literature. We followed guidelines to conducting and reporting scoping reviews [30].

### Search strategy

The current review was part of a programme of work commissioned from the National Institute for Health Research Mental Health Policy Research Unit to inform policy on services for CEN. This programme of work included evidence reviews and studies of stakeholder views and experiences, and was supported by a working group that included people with relevant lived experience of using services and clinicians from a range of disciplines and service contexts.

The programme included four individual (systematic) reviews, for which we used a single overall search strategy which was developed in collaboration with the working group of researchers, clinicians, people with relevant lived experience, and an information scientist with experience in mental health. Of the four reviews two synthesised qualitative evidence on service user experience of community mental health care for CEN [10] and clinician perspectives on what constitutes good practice, and what helps or prevents it being achieved, in community mental health services for CEN [20]. The third review evaluated international guidance regarding community service delivery and organisation for CEN [31]. The protocol for the wider programme of work was prospectively registered (CRD42019131834). This review, which constitutes the fourth part of the programme, follows the PRISMA guidelines [32] and the specific protocol for this scoping review was also registered on PROSPERO (CRD42019143165). This protocol originally encompassed a meta-analysis of quantitative data, however, the extent and heterogeneity of important literature led to a decision to conduct such analyses on a more limited subset of data. This will be reported in a separate paper.

We conducted a comprehensive search of MEDLINE (Ovid), Embase (Ovid), HMIC (Ovid), Social Policy and Practice (Ovid), CINAHL (EBSCO), and ASSIA (ProQuest), from database inception to December 2019. Search terms included terms relating to CEN, community/outpatient setting, and psychological or psychosocial treatments. An update search was conducted in November 2020 (PB). The search strategy was supplemented with a reference search of relevant systematic reviews following the original and updated search. Forward and backward citation searches using Web of Science were also performed for all included papers. No limits were placed on the language or country. Details of the search strategy are available in Additional file 1: Appendix 1.

### Study selection

All titles and abstracts were independently screened by a team of 12 people. Reliability was ensured by double checking the first 100 articles screened by each person, and a random 10% of all results were double screened by a senior researcher (LSR). Studies not meeting inclusion criteria were excluded. Subsequently, full-text articles were screened according to the specific inclusion criteria for this review by two researchers. Unclear cases and disagreements were resolved through discussion with the wider research team, including clinical members and a senior systematic reviewer.

### Selection criteria

Studies were included if they met the following criteria:

- **Participants:** Adults (operationalised as 90% of the sample over 16 years old or mean sample age of 18 or over) in which a majority (> 50%) had received a diagnosis of “personality disorder”. In order not to exclude studies in which authors wished to avoid use of this diagnostic term, or which focused on participants who had not received a formal diagnosis, we also ran searches using search terms intended to capture difficulties comparable to those experienced by people with a “personality disorder” diagnosis, including searches for samples presenting with repeated self-harm or suicide attempts, complex trauma or complex post-traumatic stress disorder (PTSD), and emotional dysregulation or instability. Clinical members in the team were consulted to achieve a consensus on the inclusion of such papers, although the large majority of the included papers focused on participants identified by a “personality disorder” diagnosis.
- **Interventions:** Treatments with a primary focus on “personality disorder” or associated needs (as defined above), including psychotherapeutic treatments and service models, conducted in a community mental health care setting, or delivered to participants living in the community during treatment.
- **Controls:** All comparators were considered (randomised and non-randomised), and we also included before and after study designs with no specific comparator group and studies in which the primary aim was uncontrolled preliminary testing of a new or newly adapted intervention.
- **Outcomes:** Any measure of global clinical or symptom severity; psychiatric hospitalisation or emergency hospital presentations; self-harm or suicide-related outcomes; quality of life or general wellbeing; general, occupational, or social functioning (including interpersonal relations).
- **Study design:** Quantitative studies, including randomised and non-randomised comparison studies and non-controlled studies with pre-post comparisons.

**Fig. 1:**
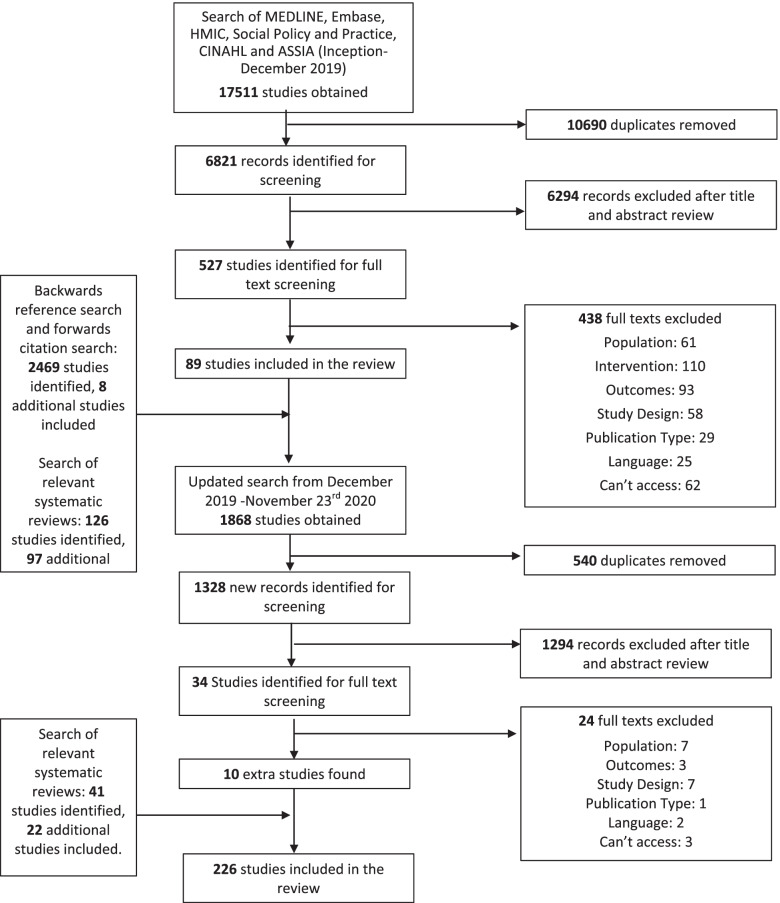
PRISMA Diagram

We excluded studies whose primary focus of treatment was not “personality disorder” diagnoses or comparable needs (as defined above), or if the treatment was conducted in forensic, crisis care, or inpatient care settings. We also excluded theses and conference abstracts. Given the very broad nature of our searches, for feasibility we included only studies published in English. The full search and screening process is depicted in Fig. 1.

### Data extraction and synthesis

Data was extracted by a team of 21 people using a standardised extraction form and was double checked for accuracy by the lead researchers. Disagreements or errors were resolved by discussion with the team including a senior member and corrected where required. Data extracted included study aims, study design, treatment and comparator details, sample characteristics and size, outcome measures, and study results. To present extracted data, papers were grouped by treatment modality, treatment/comparator category, and study design category. Treatment modality categories were developed through discussion with senior clinicians and researchers (including SP, an international expert in evaluation of psychological treatments), and included: 1) DBT; 2) cognitive and behavioural therapies; 3) MBT; 4) psychodynamic therapy; 5) schema therapy; 6) mixed modality psychotherapy; 7) other individual psychotherapy modalities; 8) social or functional orientated therapy; 9) tests of service models or service re-organisation; 10) self-management or care planning; 11) family, couple, or parenting therapies. Treatment/comparator categories included: 1) non-active or non-specialist comparator; 2) specialist or active comparator; 3) test of a modified version of the intervention; 4) test of a therapy adapted to a particular population. Study designs were categorised as follows: 1) Randomised Controlled Trials (RCTs) (noting where the study is clearly described as a pilot); 2) observational studies, including non-randomised controlled studies, and studies making pre-post comparisons within the same cohort; 3) intervention development studies. We also noted whether or not studies had an identified primary outcome.

In keeping with guidance for scoping reviews, we did not carry out quality appraisal, but have placed a greater emphasis on more robust designs in our reporting [30].

## Results

Searches of bibliographic databases returned a total of 17,511 papers of which 10,690 papers were duplicates. After screening 6,821 titles and abstracts, reviewers screened 527 full texts. 438 papers did not meet our inclusion criteria and were excluded, resulting in 88 studies included in the review. Ninety-six additional studies were identified by searching relevant systematic reviews and eight studies through reference and citation searches. The search was updated on 23/11/2020 obtaining 1,868 records. After screening 34 full texts, 10 additional studies were included in the scoping review. Overall, we identified 226 papers for inclusion (Fig. 1), reporting data from 210 distinct trials.

### Intervention types

Tables 1,2,3,4 provide summaries of included studies by intervention type, and more detailed summaries are shown in Additional file 1: Appendices 2–5. Additional file 1: Appendices 6–9 present descriptions of individual papers. There have been more studies of DBT (Table 1, Additional file 1: Appendix 2 and 6) than any other therapy modality or community-based treatment in this group (*n* = 66). We found 49 papers reporting studies of cognitive and behavioural therapies (Table 2, Additional file 1: Appendix 3 and 7), six of schema therapy (Table 2, Additional file 1: Appendix 3 and 7), 54 of psychodynamic therapy (Table 3, Additional file 1: Appendix 4 and 8), 20 of MBT (Table 3, Additional file 1: Appendix 4 and 8), ten of mixed modality psychotherapy (Table 4, Additional file 1: Appendix 5 and 9), seven of other individual psychotherapy modalities (Table 4, Additional file 1: Appendix 5 and 9), five of socially or functionally orientated therapy (Table 4, Additional file 1: Appendix 5 and 9), six of self-management or care planning (Table 4, Additional file 1: Appendix 5 and 9), and 13 tests of novel mental health service models (Table 4, Additional file 1: Appendix 5 and 9). Some studies included more than one intervention type.

**Table 1:** DBT

Study design and number of studies (n) with references	Sample size, date, and country of publication	Cohort diagnoses and demographics	Main findings
DBT vs inactive/non-specialist comparators			
RCT (*n* = 12) [33–44]	Sample size: 20–100 (*n* = 12). Date: 1990–1999 (*n* = 2); 2000–2009 (*n* = 4); 2010–2019 (*n* = 6). Country: Asia (*n* = 1); Europe (*n* = 4); North America (*n* = 4); Oceania (*n* = 1); UK (*n* = 2).	Diagnoses: “BPD” diagnosis (*n* = 10); “BPD” or “personality disorder” diagnosis and self-harm (*n* = 2). Demographics: 100% female (*n* = 5); 50–79% (*n* = 2) White.	**RCTs with primary outcomes:** On the **primary outcomes** of RCTs, compared to inactive/non-specialist controls, participants receiving DBT showed improvement in self-harm in 2/3 studies that examined self-harm (in 1 study this was only the case for clinician-rated self-harm), symptoms at discharge (1/1), global distress (0/1), hospital admissions (0/1), and “BPD” symptoms (1/1). On **non-primary outcomes**, compared to controls, participants receiving DBT showed improvement on approximately half of the outcomes. For some of the outcomes, differences were no longer significant at follow-up compared to post-treatment.
Non-randomised experiments, observational studies, quasi experiment, and natural experiment with pre-post comparison (*n* = 15) [45–59]	Sample size: < 20 (*n* = 2); 20–100 (*n* = 13). Date: 2000–2009 (*n* = 7); 2010–2019 (*n* = 8). Country: Europe (*n* = 5); North America (*n* = 8); UK (*n* = 1); Oceania (*n* = 1).	Diagnoses: “BPD”, “personality disorder”, or emotionally unstable “personality disorder” diagnosis (*n* = 11); “BPD” diagnosis and self-harm or a comorbid diagnosis (*n* = 3); severe impairment and history of suicide attempts or crisis service use (*n* = 1). Demographics: 100% female (*n* = 5); 50–79% White (*n* = 2), 80–99% White (*n* = 5).	**Non-randomised experiments:** In 1 study with two control groups, the DBT group was superior compared to TAU on the primary outcome “BPD” symptoms (1/1). In **studies with comparisons over time only**, participants improved on the one reported **primary outcome** and on close to all **secondary outcomes**. Studies focusing on patients with comorbid severe mental illness, substance dependence, or an extensive history of suicide attempts or crisis service use all showed improvement in **above-mentioned outcomes**.
Uncontrolled intervention development studies and single case study with multiple measures (*n* = 3) [60–62]	Sample size: < 20 (*n* = 1); 20–100 (*n* = 2). Date: 2000–2009 (*n* = 1); 2010–2019 (*n* = 2). Country: North America (*n* = 3).	Diagnoses: “Personality disorder” or “BPD” diagnosis (*n* = 2); severe impairment and history of suicide attempt or crisis service use (*n* = 1). Demographics: 80–99% White (*n* = 2); 100% White (*n* = 1).	**Studies with comparisons over time only:** In 1 study with a **primary outcome**, participants with severe impairment and an extensive history of suicide attempts or crisis service use improved on the primary outcome, but this was no longer significant one year later. Across studies, participants also improved on all **secondary/other outcomes**.
Implementation studies (*n* = 1) [63]	Sample size: > 100 (*n* = 1). Date: 2020 – (*n* = 1). Country: Europe (*n* = 1).	Diagnoses: “BPD” or “emotionally unstable personality disorder” diagnosis (*n *= 1). Demographics: no data reported.	**In 1 study with comparison over time only,** participants improved over time on all outcomes (1/1).
DBT vs specialist comparators			
RCT (*N* = 6) [64–69]	Sample size: 20–100 (*n* = 3); > 100 (*n* = 3). Date: 2000–2009 (*n* = 4); 2010–2019 (*n* = 2). Country: North America (*n* = 5); Oceania (*n* = 1).	Diagnoses: “BPD” diagnosis (*n* = 2); “BPD” diagnosis and self-harm (*n* = 3); “BPD” diagnosis and opiate dependence diagnosis (*n* = 1). Demographics: 100% female (*n* = 1); 50–79% White (*n* = 2), 80–99% White (*n* = 1).	**RCTs with primary outcomes:** DBT was not different or inferior to the specialist comparator in 2 RCTs (including a follow-up study) on the **primary outcomes** (suicidal episodes (0/2)). In 1 RCT, DBT was superior to the specialist comparator on the primary outcome (suicide attempts (1/1)). In 1 RCT no direct comparisons were made between specialist comparators. Across RCTs, compared to controls, DBT did not show improvement on **non-primary outcomes**, except for service use (1/1).
Non-randomised experiments, observational studies, quasi experiment, and natural experiment with pre-post comparison (*n* = 2) [70, 71]	Sample size: 20–100 (*n* = 2). Date: 2010–2019 (*n* = 2). Country: Europe (*n* = 1), UK (*n* = 1).	Diagnoses: “BPD” diagnosis (*n* = 2). Demographics: 50–79% White (*n* = 1).	**Non-randomised experiments without primary outcomes:** Participants in the DBT group showed no improvement compared to controls in the MBT group on any outcome. Participants in the combined DBT group showed no improvement compared to controls in the individual DBT group on outcomes.
Studies of partial/modified DBT			
RCT (*n* = 6 including 1 pilot) [72–77]	Sample size: 20–100 (*n* = 6). Date: 2000–2009 (*n* = 1); 2010–2019 (*n* = 5). Country: Asia (*n* = 1); Europe (*n* = 3); North America (*n* = 2).	Diagnoses: “BPD” diagnosis (*n* = 4); “BPD” diagnosis and self-harm (*n* = 2). Demographics: 100% female (*n* = 6); 50–79% White (*n* = 2); 100% White (*n* = 2).	**RCTs without primary outcomes:** In 3 RCTs, there was no difference between the adapted versions of DBT on all or most outcomes. In 3 other RCTs, compared to controls, participations receiving (adapted) DBT showed improvements on all or most outcomes.
Non-randomised experiments, observational studies, quasi experiment, and natural experiment with pre-post comparison (*n* = 10) [78–87]	Sample size: < 20 (*n* = 2); 20–100 (*n* = 6); > 100 (*n* = 2). Date: 2000–2009 (*n* = 4); 2010–2019 (*n* = 2); 2020 – (*n* = 4). Country: Europe (*n* = 2); North America (*n* = 3); Oceania (*n* = 3); Republic of Ireland and Northern Ireland (*n* = 1); UK (*n* = 1).	Diagnoses: “BPD” diagnosis and/or experiencing emotional dysregulation (*n* = 8); self-harm (*n* = 1); “BPD” and self-harm (*n* = 1) Demographics: 100% female (*n* = 2); 80–99% White (*n* = 1).	In 1 **non-randomised experiment**, compared to standard DBT, participants in the DBT skills training group showed no improvement in “BPD” symptoms, symptom severity, and suicidality (0/1). In **studies with comparison over time only**, participants improved on the **primary outcome** service use (1/1) and **most secondary outcomes**.
Uncontrolled intervention development studies and single case study with multiple measures (*n* = 3) [88–90]	Sample size: < 20 (*n* = 2); 20–100 (*n* = 1). Date: 2000–2009 (*n* = 1); 2010–2019 (*n* = 2). Country: Europe (*n* = 1); Oceania (*n* = 2).	Diagnoses: “BPD” or cluster B diagnosis (*n* = 3). Demographics: no data reported.	In 3 **studies with comparisons over time only,** participants improved on all secondary/other outcomes, except of anxiety symptoms (0/1).
Studies of adapted DBT			
RCT (*n* = 3 including 1 pilot) [91–93]	Sample size: 20–100 (*n* = 2); > 100 (*n* = 1). Date: 2010–2019 (*n* = 2); 2020- (*n* = 1). Country: Asia (*n* = 1); Europe (*n* = 1); North America (*n* = 1).	Diagnoses: “BPD” diagnosis (*n* = 1); “BPD” diagnosis/criteria and PTSD diagnosis (*n* = 2). Demographics: 100% female (*n* = 2); 80–99% White (*n* = 1); 100% male, 18–50-year-olds and married (*n* = 1).	**RCTs with primary outcomes:** In 1 RCT, compared to Cognitive Processing Therapy (CPT), participants with comorbid PTSD receiving DBT-PTSD showed improvement on **primary outcomes** (diagnostic and symptom remission of PTSD: 1/1) and secondary outcomes. In 1 RCT focusing on married men, compared to waitlist controls, participants receiving Couple-DBT showed improvement in all outcomes. One RCT did not report significance results.
Non-randomised experiments, observational studies, quasi experiment, and natural experiment with pre-post comparison (*n* = 3) [94–96]	Sample size: 20–100 (*n* = 2); > 100 (*n* = 1). Date: 2010–2019 (*n* = 3). Country: Europe (*n* = 2); Oceania (*n* = 1).	Diagnoses: “BPD” diagnosis (*n* = 2); “BPD” and eating disorder diagnosis (*n* = 1). Demographics: 100% female (*n* = 2); only 18–25-year-olds (*n* = 1); only primary caregivers of child younger than 3-years-old (*n* = 1).	**Non-randomised experiments:** In 1 study, compared to CBT, participants with a comorbid eating disorder receiving DBT showed improvement on some **primary outcomes** and most **secondary outcomes**. In 1 study, compared to the general DBT group, participants in the young adult only DBT group showed improvement in **non-primary** symptom **outcomes**. In 1 **study with comparisons over time only,** participants, i.e. caregivers of young children, improved on all outcomes.
Uncontrolled intervention development studies and single case study with multiple measures (*n* = 2) [97, 98]	Sample size: < 20 (*n* = 1); 20–100 (*n* = 1). Date: 2010–2019 (*n* = 2). Country: Europe (*n* = 2).	Diagnoses: “BPD” diagnosis or criteria (*n* = 2). Demographics: 100% female (*n* = 2); only 18–25-year-olds (*n* = 1).	**In studies with comparisons over time only**, participants improved over time on all outcomes.

**Table 2:** Cognitive and behavioural and schema therapies

Study design and number of studies (n) with references	Sample size, date, and country of publication	Cohort diagnoses and demographics	Main findings
Cognitive and behavioural treatment vs inactive/non-specialist comparators			
RCT (*n* = 18 including 4 pilot) [99–116]	Sample size: 20–100 (*n* = 12); > 100 (*n* = 6). Date: 1990–1999 (*n* = 2); 2000–2009 (*n* = 7); 2010–2019 (*n* = 9). Country: Europe (*n* = 4); North America (*n* = 6); Oceania (*n* = 1); UK (*n* = 7).	Diagnoses: “BPD” or other “personality disorder” diagnoses/criteria (*n* = 13); mixed clinical diagnoses including “personality disorder” diagnosis (*n* = 1); “BPD” diagnosis/criteria and recent or previous (repeated) self-harm (*n* = 3); recent and previous self-harm (*n* = 1). Demographics: 100% female (*n* = 4); 0–49% White (*n* = 1), 80–99% White (*n* = 5); 100% White (*n* = 5).	**RCTs with primary outcomes:** On the **primary outcomes** of RCTs, compared to controls, a greater proportion of participants receiving cognitive and behavioural therapies recovered on symptoms (1/1) and also showed improvement in “personality disorder” symptoms (3/3), symptom severity (1/2), and social functioning (1/2), but not depressive (0/1) or (social) anxiety symptoms (0/1), service use (0/1), or frequency/number of participants with self-harming/suicidal behaviour (0/4). On **non-primary outcomes**, compared to controls, participants receiving cognitive and behavioural therapies showed improvement in approximately half of the outcomes.
Non-randomised experiments, observational studies, quasi experiment, and natural experiment with pre-post comparison (*n* = 8) [117–124]	Sample size: < 20 (*n* = 1); 20–100 (*n* = 7). Date: 1990–1999 (*n* = 1); 2000–2009 (*n* = 2); 2010–2019 (*n* = 5). Country: Europe (*n* = 2); North America (*n* = 3); UK (*n* = 3).	Diagnoses: “BPD” (*n* = 3) or avoidant “personality disorder” diagnosis (*n* = 1); “BPD” diagnosis/criteria, mood disorder, history of self-harm, with our without emotional and behavioural dysregulation (*n* = 3); childhood sexual abuse (*n* = 1). Demographics: 100% female (*n* = 1); 50–79% White (*n* = 1); 80–99% White (*n* = 1).	In **studies with comparisons over time only**, participants improved on the one reported **primary outcome** (self-harm: 1/1) and most **secondary outcomes**.
Uncontrolled intervention development studies and single case study with multiple measures (*n* = 11) [125–135]	Sample size: < 20 (*n* = 8); 20–100 (*n* = 3). Date: 2000–2009 (*n* = 2); 2010–2019 (*n* = 9). Country: Asia (*n* = 2); Europe (*n* = 3); North America (*n* = 1); Oceania (*n* = 1); UK (*n* = 4).	Diagnoses: “BPD” (*n* = 4) or other “personality disorder” diagnoses (*n* = 4); “BPD” diagnosis/features and comorbid mood disorder (*n* = 2) or drug/alcohol disorder (*n* = 1). Demographics: 80–99% White (*n* = 1); 100% White (*n* = 1); older age (*n* = 1).	In **studies with comparisons over time only**, participants improved over time on the **primary outcomes** symptoms/distress (2/2) and quality of life (1/1), and also showed no dropouts (1/1). Participants improved on **secondary outcomes**. Patients with a current substance misuse disorder showed a reduction in drug use (1/1). Elderly patients with a chronic mood or adjustment disorder improved in symptom distress (1/1) and some but not all aspects of schema and coping variables (1/1).
Cognitive and behavioural treatment vs specialist comparators			
RCT (*n* = 4) [136–139]	Sample size: 20–100 (*n* = 4). Date: 2000–2009 (*n* = 3), 2010–2019 (*n* = 1). Country: Europe (*n* = 3); Europe and North America (*n* = 1).	Diagnoses: “BPD” features/diagnosis (*n* = 2) or other “personality disorder” diagnosis (*n* = 2) Demographics: 100% White (*n* = 1).	**RCTs with primary outcomes:** In 3 RCTs comparing cognitive and behavioural therapy with specialist comparators, there were no between-group differences on **primary outcomes** (symptom improvement: 0/1; symptoms severity: 0/1; interpersonal problems: 0/1) or **secondary outcomes**. In 1 RCT, significantly more participants receiving Schema Focused Therapy (SFT) recovered on the **primary outcome** (“BPD” symptoms: 1/1) as well as on three **secondary outcomes** compared to cognitive therapy.
Non-randomised experiments, observational studies, quasi experiment, and natural experiment with pre-post comparison (*n* = 3) [140–142]	Sample size: 20–100 (*n* = 1); > 100 (*n* = 2). Date: 2000–2009 (*n* = 1); 2010–2019 (*n* = 2). Country: Europe (*n* = 3).	Diagnoses: “Personality disorder” diagnosis (*n* = 2); cluster B “personality disorder” diagnosis with comorbid Axis I disorder (*n* = 1). Demographics: no data report.	In 3 **non-randomised experiments**, there were no differences between the cognitive behavioural treatment and specialist comparators on **primary outcomes** (personality functioning: 0/1; symptom severity: 0/1) and all or most **secondary outcomes** (0/3).
Uncontrolled intervention development studies and single case study with multiple measures (*n* = 1) [143]	Sample size: < 20 (*n* = 1). Date: 2010–2019 (*n* = 1). Country: North America (*n* = 1).	Diagnoses: NSSI disorder (*n* = 1). Demographics: 50–79% White (*n* = 1).	**One study with comparisons over time only** did not report significant results for outcomes on patients with NSSI disorder. However, 8/10 participants reported meaningful reductions in self-harming behaviour.
Studies of modified cognitive and behavioural treatments			
RCT (*n* = 2 including 1 pilot) [144, 145]	Sample size: < 20 (*n* = 1); 20–100 (*n* = 1). Date: 1990–1999 (*n* = 1); 2010–2019 (*n* = 1). Country: North America (*n* = 1); UK (*n* = 1).	Diagnoses: “BPD” diagnosis (*n* = 1); previous suicide attempts, antidepressants taken as part of an overdose, and suicidal behaviour (*n* = 1). Demographics: 80–99% White (*n* = 1).	**RCTs with primary outcomes:** On the **primary outcome** of 1 RCT, findings for differences between the cognitive Behavioural Problem Solving and TAU group on suicidality were mixed (0/1). Findings were mixed or showed no between-group differences for **non-primary outcomes** (0/2).
Non-randomised experiments, observational studies, quasi experiment, and natural experiment with pre-post comparison (*n* = 1) [146]	Sample size: 20–100 (*n* = 1). Date: 2000–2009 (*n* = 1). Country: Europe (*n* = 1).	Diagnoses: “Personality disorder” diagnosis, excluding borderline, schizotypal, schizoid, antisocial, or NOS “personality disorder” diagnoses (*n* = 1). Demographics: no data reported.	The 1 study utilised a crossover design and showed significant improvements over the treatment period as a whole, but no between-group differences.
Studies of adapted cognitive and behavioural treatments			
Uncontrolled intervention development studies and single case study with multiple measures (*n* = 1) [147]	Sample size: < 20 (*n* = 1). Date: 2010–2019 (*n* = 1). Country: Oceania (*n* = 1).	Diagnoses: “Personality disorder” diagnosis (*n* = 1). Demographics: no data reported.	In 1 **study with comparisons over time only**, no statistical analysis was conducted. However, 5/8 patients no longer met criteria for an avoidant “personality disorder” at end of follow-up.
Schema therapy vs inactive/non-specialist comparators			
RCT (*n* = 1) [148]	Sample size: > 100 (*n* = 1). Date: 2010–2019 (*n* = 1). Country: Europe (*n* = 1).	Diagnoses: Avoidant, dependent, obsessive–compulsive, paranoid, histrionic, or narcissistic “personality disorder” diagnosis (*n* = 1). Demographics: no data reported.	On the **primary outcome** of the 1 RCT, compared to controls, a greater proportion of participants receiving schema therapy recovered (1/1). Compared to controls, participants also improved on some **non-primary outcomes**.
Uncontrolled intervention development studies and single case study with multiple measures (*n* = 4) [149–152]	Sample size: < 20 (*n* = 4). Date: 2000–2009 (*n* = 1); 2010–2019 (*n* = 3). Country: Europe (*n* = 3); North America (*n* = 1).	Diagnoses: “BPD” (*n* = 3) or other “personality disorder” diagnosis (*n* = 1). Demographics: 100% female (*n* = 3); old age (*n* = 1).	In the 1 study that reported significant results **with comparisons over time only**, participants improved on “BPD” symptoms (1/1) and most other outcomes.
Studies of modified schema therapy			
RCT (*n* = 1) [153]	Sample size: 20–100 (*n* = 1). Date: 2000–2009 (*n* = 1). Country: Europe (*n* = 1).	Diagnoses: “BPD” diagnosis (*n* = 1). Demographics: 80–99% (*n* = 1).	**RCTs with primary outcomes:** On the **primary outcome** of 1 RCT, there was no difference between participants receiving schema therapy with and those without phone support on recovery from “BPD” (0/1). There was also no significant difference on **non-primary outcomes** (0/1).

**Table 3:** Psychodynamic and MBT studies

Study design and number of studies (N) with references	Sample size, date, and country of publication	Cohort diagnoses and demographics	Main findings
MBT vs inactive/non-specialist comparators			
RCT (*n* = 4) [43, 154–156]	Sample size: 20–100 (*n* = 4). Date: 1990–1999 (*n* = 1); 2000–2009 (*n* = 2); 2010–2019 (*n* = 1). Country: Asia (*n* = 1); UK (*n* = 3).	Diagnoses: “BPD” diagnosis (*n* = 4). Demographics: no data reported.	**RCTs with primary outcomes:** In the **primary outcomes** of 2 RCTs, compared to controls, participants receiving MBT showed improvement in the proportion of patients making suicide attempts (1/1) and in “BPD” symptoms (1/1). Compared to controls, participants receiving MBT showed improvement in all **non-primary outcomes**.
Non-randomised experiments, observational studies, quasi experiment, and natural experiment with pre-post comparison (*n* = 6) [157–162]	Sample size: < 20 (*n* = 2); 20–100 (*n* = 3); > 100 (*n* = 1). Date: 2010–2019 (*n* = 6). Country: Europe (*n* = 6).	Diagnoses: “BPD” (*n* = 4) or other “personality disorder” diagnosis (*n* = 1); “personality disorder” diagnosis and poor functioning (*n* = 1). Demographics: 100% female (*n* = 1).	In 1 **non-randomised experiments**, compared to controls, participants improved on some **non-primary outcomes.** In **studies with comparisons over time only**, participants showed improvements on all **primary and non-primary outcomes**.
MBT vs specialist comparators			
RCT (*n* = 4) [163–166]	Sample size: 20–100 (*n* = 1); > 100 (*n* = 3). Date: 2000–2009 (*n* = 1); 2010–2019 (*n* = 3). Country: Europe (*n* = 3); UK (*n* = 1).	Diagnoses: “BPD” diagnosis (*n* = 3); “BPD” and suicide attempt or life-threatening self-harm (*n* = 1). Demographics: 50–79% White (*n* = 1).	**RCTs with primary outcomes:** In the **primary outcomes** of RCTs, compared to specialist controls, participants receiving MBT showed improvement in suicidal behaviours (1/1) and number of hospitalisations (1/1), but not in “borderline symptoms” (0/1). Compared to specialist comparators, participants receiving MBT did not show improvements in most **non-primary outcomes**.
Non-randomised experiments, observational studies, quasi experiment, and natural experiment with pre-post comparison (*n* = 5) [70, 167–170]	Sample size: 20–100 (*n* = 4); > 100 (*n* = 1). Date: 2010–2019 (*n* = 5). Country: Europe (*n* = 1), UK (*n* = 4).	Diagnoses: “BPD” (*n* = 2) or “personality disorder” diagnosis (*n* = 3). Demographics: 50–79% White (*n* = 1); 80–99% White (*n* = 3).	In **non-randomised studies**, compared to an alternative psychoanalytic model, the MBT group did not significantly improve on the **primary outcome** of bed use (0/1). Compared to specialist treatments, participants receiving MBT did not show improvements in more than half of **non-primary outcomes.** In **2 studies with comparisons over time only**, participants improved on less than half of the outcomes.
Studies of modified MBT			
RCT (*n* = 1) [171]	Sample size: > 100 (*n* = 1). Date: 2020- (*n* = 1). Country: Europe (*n* = 1).	Diagnoses: “Personality disorder” diagnosis (*n* = 1). Demographics: no data reported.	**RCTs with primary outcomes:** Compared to lower intensity outpatient MBT, higher intensity day hospital MBT showed no difference in the **primary outcome** of symptom severity and **non-primary outcomes**.
Psychodynamic treatment vs inactive/non-specialist comparators			
RCT (*n* = 6) [109, 172–176]	Sample size: 20–100 (*n* = 4); > 100 (*n* = 2). Date: 1990–1999 (*n* = 2); 2000–2009 (*n* = 3); 2010–2019 (*n* = 1). Country: Europe (*n* = 3); North America (*n* = 3).	Diagnoses: “BPD” (*n* = 1) or other “personality disorder” diagnosis (*n* = 4); long term psychiatric difficulties disrupting functioning (*n* = 1). Demographics: no data reported.	**RCTs with primary outcomes:** In the **primary outcomes** of RCTs, compared to controls, participants receiving psychodynamic therapy showed improvement in symptom severity (2/2), social functioning (1/2), and interpersonal functioning (1/1), but not dysfunctional “borderline beliefs” (0/1), anxiety symptoms (0/1), or the number of participants meeting diagnostic criteria for a “personality disorder” diagnosis (0/1). Compared to controls, participants receiving psychodynamic therapy improved on **most non-primary outcomes**.
Non-randomised experiments, observational studies, quasi experiment, and natural experiment with pre-post comparison (*n* = 26) [48, 177–201]	Sample size: < 20 (*n* = 1); 20–100 (*n* = 18); > 100 (*n* = 7). Date: 1990–1999 (*n* = 6); 2000–2009 (*n* = 12); 2010–2019 (*n* = 7); 2020- (*n* = 1). Country: Australia (*n* = 7); Europe (*n* = 10); North America (*n* = 6); UK (*n* = 3).	Diagnoses: “Personality disorder” (*n* = 11) or “BPD” diagnosis/criteria (*n* = 8); “personality disorder” diagnosis and comorbid Axis I mental health problems (*n* = 3); treatment resistant depression with comorbid “personality disorder” and childhood trauma (*n* = 1); “personality disorder” diagnosis and poor interpersonal functioning (*n* = 2); poor interpersonal functioning (*n* = 1). Demographics: 100% female (*n* = 1); 80–99% White (*n* = 3); 100% White (*n* = 1).	In **non-randomised experiments**, participants showed improvements compared to controls on the following **primary measures:** reflective functioning (2/2), “personality disorder” symptoms (1/1), social functioning (1/1), and depressive symptoms (1/1). Compared to controls, participants improved on almost all **non-primary outcomes.** In **studies with comparisons over time only**, participants improved in all **primary outcomes** in interpersonal functioning (3/3) and symptom severity (1/1) and close to all **non-primary outcomes**.
Uncontrolled intervention development studies and single case study with multiple measures (*n* = 1) [202]	Sample size: 20–100 (*n* = 1). Date: 2000–2009 (*n* = 1). Country: North America (*n* = 1).	Diagnoses: “BPD” symptoms and suicidal or self-injurious behaviour (*n* = 1). Demographics: 100% female (*n* = 1); > 50% White (*n* = 1).	**Studies with comparisons over time only:** One uncontrolled feasibility trial found that patients given psychodynamic therapy improved over time on outcomes (1/1).
Psychodynamic treatment vs specialist comparators			
RCT (*n* = 8) [67, 138, 139, 203–207]	Sample size: 20–100 (*n* = 8) Date: 1990–1999 (*n* = 1); 2000–2009 (*n* = 2); 2010–2019 (*n* = 5). Country: Europe (*n* = 5); Europe and North America (*n* = 1); North America (*n* = 2).	Diagnoses: “BPD” (*n* = 5) or other “personality disorder” diagnosis (*n* = 3). Demographics: 50–79% White (*n* = 1); 80–99% White (*n* = 2); 100% White (*n* = 1).	**RCTs with primary outcomes:** In **primary outcomes** of RCTs, compared to cognitive therapy, participants receiving psychodynamic therapy did not significantly improve in symptom severity (0/1). In 1/3 RCTs, compared to General Psychiatric Management, participants receiving psychodynamic therapy made significantly more overall progress in therapy overall. Compared to specialist controls, participants receiving psychodynamic therapy did not show improvements on almost any **non-primary outcomes**. One RCT did not make direct comparisons between groups.
Non-randomised experiments, observational studies, quasi experiment, and natural experiment with pre-post comparison (*n* = 4) [208–211]	Sample size: 20–100 (*n* = 3); > 100 (*n* = 1). Date: 1990–1999 (*n* = 1); 2010–2019 (*n* = 3). Country: Europe (*n* = 3); North America (*n* = 1).	Diagnoses: “BPD” (*n* = 2) or other “personality disorder” diagnosis (*n* = 1); “personality disorder” diagnosis with or without comorbid substance misuse (*n* = 1). Demographics: no data reported.	In **1 non-randomised experiment,** compared to DBT, participants given Dynamic Deconstructive Psychotherapy had significantly greater improvement in the **primary outcome** of symptom severity (1/1). Compared to controls, participants improved on all or **most non-primary outcomes**. In 1 **study with comparisons over time only,** patients with and without comorbid substance misuse improved on outcomes.
Comparisons of psychodynamic treatment settings			
Non-randomised experiments, observational studies, quasi experiment, and natural experiment with pre-post comparison (*n* = 6) [141, 212–216]	Sample size: > 100 (*n* = 6). Date: 2000–2009 (*n* = 2); 2010–2019 (*n* = 4). Country: Europe (*n* = 3), UK (*n* = 2); Europe and UK (*n* = 1).	Diagnoses: “Personality disorder” diagnosis (*n* = 5); severe “personality disorder” diagnosis (*n* = 1). Demographics: no data reported.	**Six non-randomised experiments** compared psychodynamic treatment in varying contexts. There were no significant differences between day hospital, outpatient, and inpatient services on the **primary outcome** (symptom severity) or **non-primary outcomes**. Community or step-down services resulted in significantly improved **non-primary outcomes** compared to residential services.
Studies of adapted psychodynamic treatment			
RCT (*n* = 2) [217, 218]	Sample size: 20–100 (*n* = 2). Date: 2000–2009 (*n* = 1); 2010–2019 (*n* = 1). Country: North America (*n* = 2).	Diagnoses: “BPD” diagnosis and alcohol use or substance dependence (*n* = 2). Demographics: no data reported.	**RCTs with primary outcomes:** In the **primary outcomes** of RCTs, comparing Dynamic Deconstructive Psychotherapy combined with alcohol rehabilitation to TAU with alcohol rehabilitation for patients with co-occurring substance use disorders, Dynamic Deconstructive Psychotherapy patients showed significantly greater clinically meaningful improvement (1/1) and improved in alcohol misuse (1/1) and use of institutional care (1/1). Participants receiving Dynamic Deconstructive Psychotherapy showed significant improvements in more than half of **non-primary outcomes** compared to TAU.
Non-randomised experiments, observational studies, quasi experiment, and natural experiment with pre-post comparison (*n* = 1) [219]	Sample size: 20–100 (*n* = 1). Date: 2011–2019 (*n* = 1). Country: Europe (*n* = 1).	Diagnoses: “BPD” diagnosis (*n* = 1). Demographics: relatively low socio-economic status (*n* = 1).	**In 1 non-randomised experiment,** a brief psychoeducational program based on General Psychiatric Management was more effective than generic outpatient treatment (1/1).

**Table 4:** Other studies

Study design and number of studies (N) with references	Sample size, date, and country of publication	Cohort diagnoses and demographics	Main findings
Mixed therapeutic modalities vs inactive/non-specialist comparators			
RCT (*n* = 3) [220–222]	Sample size: 20–100 (*n* = 2); > 100 (*n* = 1) Date: 2010–2019 (*n* = 3) Country: Europe (*n* = 3)	Diagnoses: “BPD” diagnosis (*n* = 3). Demographics: 100% female (*n* = 1)	**RCTs with primary outcomes:** On the **primary outcomes** of RCTs, compared to controls, fewer participants in the intervention group dropped out (1/1) and attempted suicide (1/1), but there was no between-group difference in “BPD” symptoms (0/1). Compared to controls, participants in the intervention group showed greater improvement in most **non-primary outcomes**
Non-randomised experiments, observational studies, quasi experiment, and natural experiment with pre-post comparison (*n* = 6) [223–228]	Sample size: 20–100 (*n* = 3); > 100 (*n* = 3) Date: 1990–1999 (*n* = 1); 2000–2009 (*n* = 3); 2010–2019 (*n* = 2) Country: Europe (*n* = 5); North America (*n* = 1)	Diagnoses: “BPD” (*n* = 1) or other “personality disorder” diagnosis (*n* = 4); “personality disorder” diagnosis with self-harm, suicidal, or impulsive behaviour (*n* = 1). Demographics: not reported	In **studies with comparisons over time only**, participants improved on following **primary outcomes**: “BPD” symptoms (1/1), symptom distress, interpersonal relations and social functioning (1/1), and service use (1/1), as well as **non-primary outcomes**
Mixed therapeutic modalities vs specialist comparators			
RCT (*n* = 1) [229]	Sample size: > 100 (*n* = 1) Date: 2010–2019 (*n* = 1) Country: Europe (*n* = 1)	Diagnoses: “Personality disorder” diagnosis (*n* = 1). Demographics: not reported	**RCTs with primary outcomes:** In the 1 **RCT**, cost-effectiveness did not differ between the step-down treatment and outpatient control group (0/1)
Other individual therapy vs inactive/non-specialist comparators			
RCT (*n* = 5 including 1 pilot. 1 also reported in specialist comparators) [175, 230–233]	Sample size: 20–100 (*n* = 4); > 100 (*n* = 1) Date: 1990–1999 (*n* = 1); 2000–2009 (*n* = 1); 2010–2019 (*n* = 3) Country: Europe (*n* = 3); North America (*n* = 2)	Diagnoses: “BPD” (*n* = 1) or other “personality disorder” diagnosis (*n* = 3); “BPD” diagnosis and major depressive disorder (*n* = 1) Demographics: 100% female (*n* = 2); 50–79% White (*n* = 1)	**RCTs with primary outcomes:** In 1 RCT, compared to TAU, participants with “BPD” and major depressive disorder receiving Abandonment psychotherapy improved on the **primary outcomes** (suicidal relapse: 1/1; hospitalisation: 1/1) and **non-primary outcomes**. In 1 RCT, there was no difference between the immediate and delayed psychoeducation group on the **primary outcome** (“BPD” severity: 0/1). In 1 RCT, compared to Group Psychotherapy, participants receiving Body-Awareness Group Therapy showed improvement in all **non-primary outcomes**. In 1 RCT, compared to waitlist controls, participants receiving Brief Adaptive Psychotherapy and Psychodynamic Psychotherapy showed improvement in all **non-primary outcomes**
Non-randomised experiments, observational studies, quasi experiment, and natural experiment with pre-post comparison (*n* = 1) [234]	Sample size: 20–100 (*n* = 1). Date: 2010–2019 (*n* = 1) Country: North America (*n* = 1)	Diagnoses: Adverse childhood experiences (*n* = 1) Demographics: 50–79% White (*n* = 1)	In 1 study **with comparisons over time only**, participants with adverse childhood experiences improved on all outcomes
Other individual therapy vs specialist comparators			
RCT (*n* = 2 including 1 also reported in non-specialist) [67, 231]	Sample size: 20–100 (*n* = 1); > 100 (*n* = 1) Date: 2000–2009 (*n* = 1); 2010–2019 (*n* = 1) Country: Europe (*n* = 1); North America (*n* = 1)	Diagnoses: “BPD” diagnosis (*n* = 1); “BPD” diagnosis and major depressive disorder (*n* = 1). Demographics: 50–79% White (*n* = 1)	**RCTs with primary outcomes:** In 1 RCT focusing on patients with major depressive disorder and “BPD”, there was no difference between Abandonment psychotherapy and TAU on the **primary outcome** (suicidal relapse: 0/1) and **non-primary outcomes**. Though no direct contrasts were made, in one RCT of DBT, supportive treatment, and psychodynamic therapy, participants receiving supportive treatment improved on some **non-primary outcomes**
Social-interpersonal and functional therapies vs non-specialist/inactive comparators			
RCT (*n* = 3) [235–237]	Sample size: 20–100 (*n* = 1); > 100 (*n* = 2) Date: 1990–1999 (*n* = 1); 2000–2009 (*n* = 1); 2010–2019 (*n* = 1) Country: Europe (*n* = 1); North America (*n* = 1); UK (*n* = 1)	Diagnoses: “Personality disorder” (*n* = 1) or “BPD” diagnosis (*n* = 2) Demographics: not reported	**RCTs with primary outcomes:** On the **primary outcomes** of RCTs, compared to controls, participants in the intervention group showed improvement in social functioning (1/1) and social problem-solving skills (1/1), but not general functioning (0/1). Compared to controls, participants in the intervention group showed greater improvement on half of the **non-primary outcomes**
Social-interpersonal and functional therapies vs specialist comparators			
RCT (*n* = 2 including 1 pilot) [238, 239]	Sample size: 20–100 (*n* = 2) Date: 1990–1999 (*n* = 1); 2020- (*n* = 1) Country: North America (*n* = 1); UK (*n* = 1)	Diagnoses: Avoidant “personality disorder” diagnosis (*n* = 1); at least 3 episodes of self-harm in the past 3 months (*n* = 1) Demographics: not reported	**RCTs with primary outcomes:** On the **primary and secondary outcomes of RCTs**, there were no significant differences between skills training in vivo and skills training in the clinic as well as between Functional Imagery Training (FIT) and delayed FIT across outcomes (0/2)
Self-management and care planning vs self-management			
RCT (*n* = 2) [240, 241]	Sample size: 20–100 (*n* = 2) Date: 2010–2019 (*n* = 2) Country: Europe (*n* = 1); UK (*n* = 1)	Diagnoses: “BPD” diagnosis and past self-harm (*n* = 1); “personality disorder” diagnosis (*n* = 1). Demographics: 50–79% White (*n* = 1); 100% White (*n* = 1)	**RCTs with primary outcomes:** On the **primary outcomes** of 1 RCT, the Joint Crisis Plan and TAU group did not differ in the frequency or proportion of participants who self-harm (0/1). In **non-primary outcomes**, compared to TAU, participants receiving Joint Crisis planning did not differ in outcomes. Compared to Structured Goal-Focused Pre-Treatment Intervention (GFPTI), participants receiving therapeutic assessment showed improvements in more than half of **non-primary outcomes**
Self-management and care planning vs established generic or specialist mental health services			
RCT (*n* = 1) [242]	Sample size: 20–100 (*n* = 1) Date: 2000–2010 (*n* = 1) Country: UK (*n* = 1)	Diagnoses: Severe mental illness and comorbid personality disorder or difficulty (*n* = 1)	**In 1 RCT with primary outcomes**, there were no differences between Nidotherapy enhanced assertive outreach and standard assertive outreach in **primary outcomes** (number of admissions: 0/1; duration of bed use: 0/1) or **non-primary outcomes**
Non-randomised experiments, observational studies, quasi experiment, and natural experiment with pre-post comparison (*n* = 3) [243–245]	Sample size: 20–100 (*n* = 2); > 100 (*n* = 1) Date: 2010–2019 (*n* = 3) Country: Europe (*n* = 1); North America (*n* = 1); UK (*n* = 1)	Diagnoses: “Personality disorder” diagnosis (*n* = 2); major depressive disorder with or without a “personality disorder” diagnosis (*n* = 1) Demographics: not reported	**Non-randomised experiments:** In 1 study, compared to TAU, participants receiving collaborative care management showed improvement on the **primary outcome** (remission of depression: 1/1). In 1 study, compared to TAU, participants in the Collaborative Care Programme (CCP) improved on one of two **non-primary outcomes** In 1 **study with comparisons over time**, participants improved on outcomes
Novel mental health service model vs day hospital			
RCT (*n* = 5) [246–250]	Sample size: 20–100 (*n* = 1); > 100 (*n* = 4) Date: 2000–2009 (*n* = 1); 2010–2019 (*n* = 4) Country: Europe (*n* = 5)	Diagnoses: “Personality disorder” (*n* = 4) or “BPD” diagnosis (*n* = 1) Demographics: not reported	**RCTs with primary outcomes:** Four RCTs reported results for the same sample at different time points. Compared to outpatient controls, participants in the step-down day hospital group showed no difference in **non-primary outcomes** at 18 months. On **primary outcomes**, compared to outpatient controls, participants in the step-down group showed less improvement in functioning (0/1) at 37 months. There were not between-group differences in social and occupational functioning (0/2), interpersonal problems (0/2), depressive symptoms (0/2), symptom severity (0/2), and quality of life (0/2) at 37 months and 6 years as well as functioning (0/1) at 6 years. There were no between-group differences in **non-primary outcomes** at 37 months and 6 years. In 1 RCT only including patients with a “BPD” diagnosis, compared to outpatient controls, participants in the step-down intervention group showed greater improvement in half of the **non-primary outcomes** at 6 years
Novel mental health service model vs established generic or specialist mental health services			
RCT (*n* = 2) [251, 252]	Sample size: > 100 (*n* = 2) Date: 2010–2019 (*n* = 2) Country: Oceania (*n* = 1); UK (*n* = 1)	Diagnoses: “BPD” (*n* = 1) or “personality disorder” diagnosis (*n* = 1) Demographics: not reported	**RCTs with primary outcomes:** On the **primary outcomes** of 1 RCT, compared to TAU, participants receiving stepped care psychological therapy showed improvement in bed days (1/1) and A&E attendance (1/1). In 1 RCT, compared TAU, participants in the democratic therapeutic community group did not differ in hospital admission (0/1), but showed greater improvement in some **non-primary outcomes**
Non-randomised experiments, observational studies, quasi experiment, and natural experiment with pre-post comparison (*n* = 5) [253–257]	Sample size: 20–100 (*n* = 2); > 100 (*n* = 3) Date: 2010–2019 (*n* = 5) Country: North America (*n* = 1); Oceania (*n* = 1); UK (*n* = 3)	Diagnoses: “Personality disorder” (*n* = 4) or “BPD” diagnosis (*n* = 1). Demographics: 50–79% White (*n* = 1)	In **studies with comparisons over time only**, participants improved on most outcomes
Uncontrolled intervention development studies and single case study with (*n* = 1) [258]	Sample size: < 20 (*n* = 1) Date: 2010–2019 (*n* = 1) Country: UK (*n* = 1)	Diagnoses: “Personality disorder” diagnosis (*n* = 1). Demographics: older adults, + 65 (*n* = 1)	In 1 **intervention study with comparisons over time only,** there was some evidence for improvement on outcomes, but no statistical analysis was conducted (1/1)

**Fig. 2:**
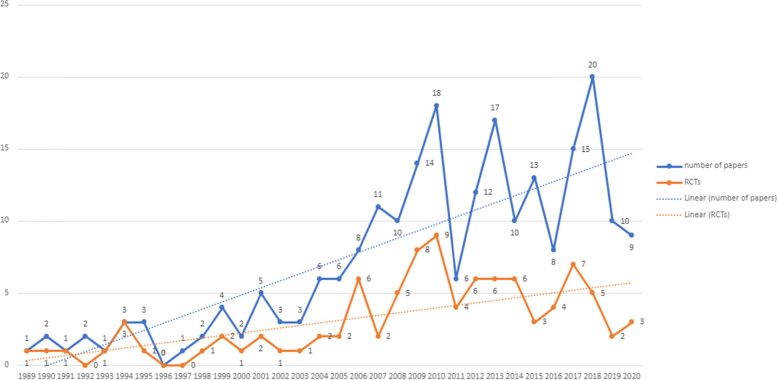
Number of Papers by Year

**Fig. 3:**
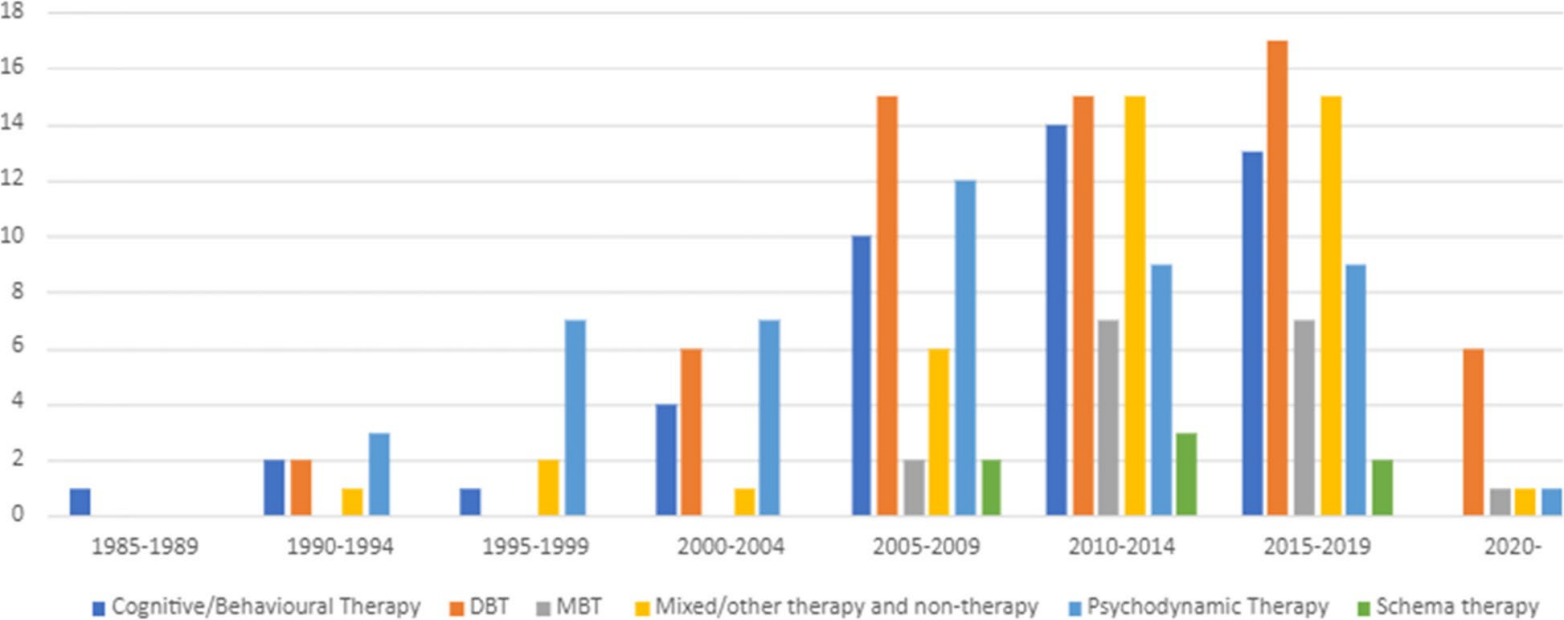
Number of Papers by Treatment Type and by Year

Included papers were published between 1989 and 2020. As shown in Fig. 2, there has been a progressive increase in papers over this time, with both the number of RCTs and other study designs increasing from a very small number per year in the 1990s, to 10–20 per year from 2010 onwards. However, the dearth of studies of any type prior to 2000 and the slow rate of increase in numbers of RCTs examining interventions for CEN are notable. As shown by Fig. 3, studies testing psychodynamic therapy were the most frequent until 2005, with studies of cognitive and behavioural therapies and DBT becoming the most prevalent in the last 15 years. There has also been an increase in the number of studies evaluating mixed therapeutic approaches over time. However, the number of studies exploring service models has remained very low (*n* = 13; 2010 to 2019) (*see* Table 4).

### Locations of interventions

**Fig. 4:**
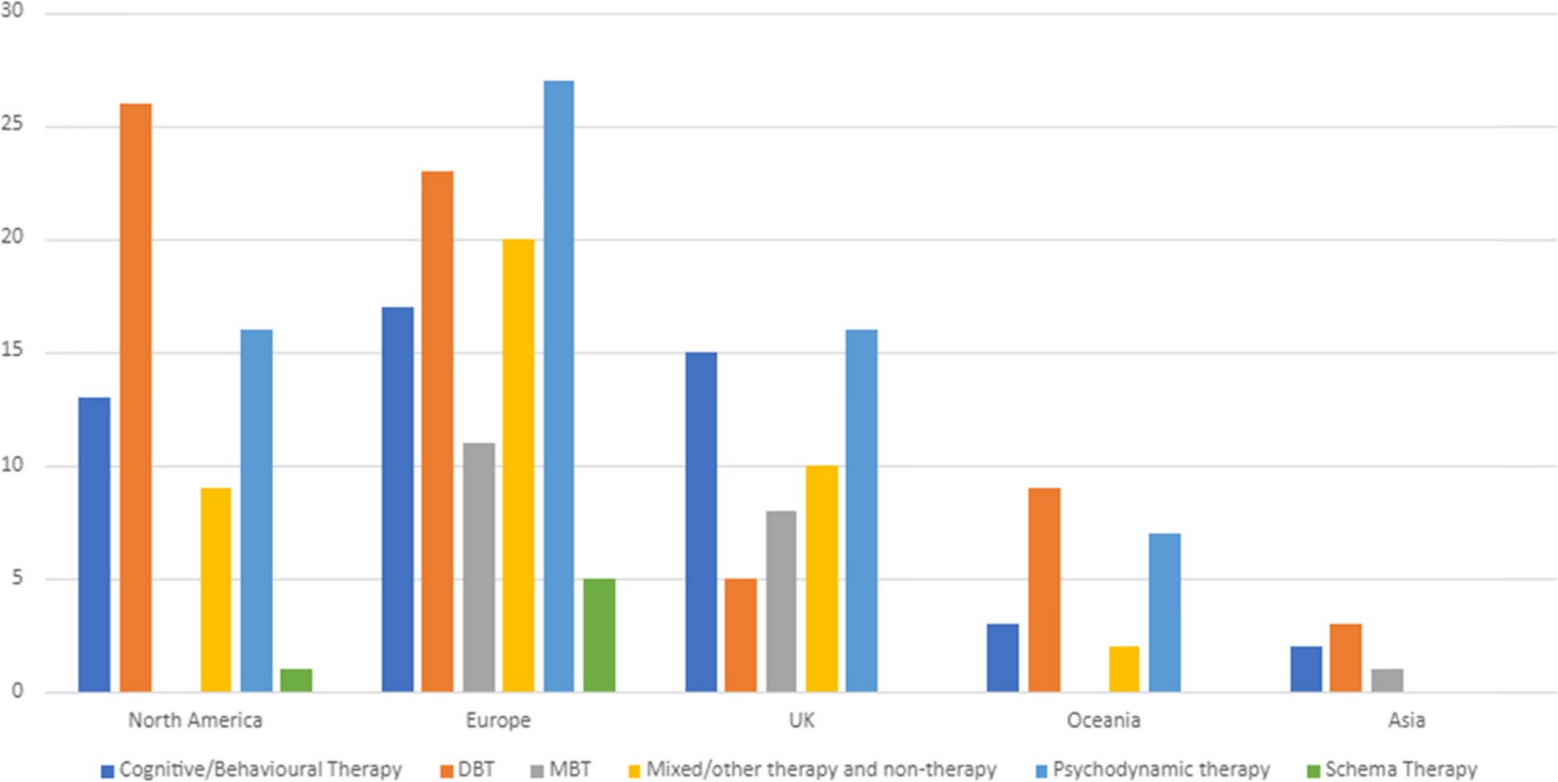
Locations of Interventions

Studies were conducted in a range of countries across Asia (*n* = 6), Europe (European countries other than the UK) (*n* = 98), North America (*n* = 60), Oceania (*n* = 21), and the UK (*n* = 43) (Fig. 4). Two studies were conducted in more than one continent. DBT studies made up around half of all studies conducted in North America (*n* = 26) and Oceania (*n* = 9), but a much smaller proportion in Europe (*n* = 22), the UK (*n* = 5), and Asia (*n* = 3). Cognitive and behavioural and schema therapy studies made up around a third or more of studies in Asia (*n* = 2) and the UK (*n* = 15), but a lower proportion in Europe (*n* = 22), North America (*n* = 14), and Oceania (*n* = 3). Psychodynamic and MBT therapies also made up a third or more of studies in the UK (*n* = 14) as well as in Oceania (*n* = 7) and Europe (*n* = 38), but a lower proportion elsewhere (Asia *n* = 1; North America *n* = 16). Studies exploring other types of treatment were mainly conducted in Europe (*n* = 20), followed by the UK (*n* = 10), North America (*n* = 9), and Oceania (*n* = 2).

Study sample sizes varied from five to 9,614 and have generally increased over the last 30 years. Overall, around half to two thirds of studies of each therapeutic modality had samples between 20 and 100. Cognitive and behavioural and schema therapy studies were generally smaller (samples < 20 = 16/55; > 100 = 9/55), and psychodynamic and MBT therapies were larger (samples < 20 = 3/74; > 100 = 22/74). Sample sizes of RCTs have also risen during this period. The mean sample size rose from 55.3 (SD = 35.7) between 1990 and 1999 to 97.4 (SD = 98.1) between 2010 and 2019.

### Outcomes

Overall, “BPD” was the most studied diagnosis, with 128 studies (57%) including samples partially or wholly made up of people given a diagnosis of “BPD”, followed by studies including participants with a mixture of “personality disorder” diagnoses (*n* = 79, 35%). Fourteen (6%) studies did not have “personality disorder” diagnosis as an inclusion criterion, but used inclusion criteria that in the judgement of the team, including clinicians, appeared to encompass similar difficulties, for example focusing on repeated self-harm or suicide attempts, complex trauma or PTSD, and emotional dysregulation or instability. These studies were included in an attempt to capture studies relevant to people with CEN in which investigators had decided not to use the “personality disorder” label as a primary way of identifying participants. “BPD” was the most studied diagnosis across treatment types, except for psychodynamic therapies and other therapies, where the largest category was studies in which participants had a mixture of “personality disorder” diagnoses. Most samples of studies that reported the sex or gender and/or ethnicity of participants were mostly female and White with 39 studies including only women and 13 studies only White participants. One study included a 100% male sample. The remaining studies included mixed samples or did not report sex or gender and/or ethnicity.

Ninety-six out of 226 studies had specified primary outcomes, including 21/65 studies on DBT, 10/20 studies on MBT, 23/54 studies on psychodynamic therapy, 24/49 studies on cognitive and behavioural therapy, 5/6 studies on schema therapy, and 20/41 studies on other treatment. The most studied outcomes were improvement in overall symptom severity (approximately *N* = 106), personality symptoms/functioning/diagnosis (approx. *N* = 113), as well as other symptoms, such as anxiety, depressive, or PTSD symptoms (approx. *N* = 115). Other commonly examined outcomes were social functioning and interpersonal symptoms and problems (approx. *N* = 88), self-harm, suicide attempts, and suicidality (approx. *N* = 87), service use, such as crisis service use and length and number of hospitalisations (approx. *N* = 66), as well as quality of life (approx. *N* = 44) and general functioning (approx. *N* = 48). Approximately 145 studies also examined a range of other outcomes.

### Main findings

In the following sections we highlight the main findings for each intervention type by focusing on results of studies with an identified primary outcome, and on those with sample sizes greater than 100 participants. Findings are further described in Tables 1, 2, 3, 4 with more detailed summarise shown in Additional file 1: Appendices 2–5, including findings of uncontrolled studies which only made comparisons over time within the same subjects: the Tables indicate that these almost always showed a tendency for improvements over time. For this reason and because of their relative methodological weaknesses we do not summarise them further in the text below (unless of interest because adaptations have been made and feasibility of treatment tested for specific groups who are not usually the focus of trials, such as people with comorbidities, or older or younger people or ethnic or sexual minorities).

### DBT

Table 1, Additional file 1: Appendix 2 and Appendix 6 summarise studies investigating the effectiveness of DBT (*n* = 66), of which the largest group was RCTs (*n* = 27), followed by uncontrolled studies making only pre-post comparisons (*n* = 24), non-randomised studies with contemporaneous comparators (including quasi and natural experiments) (*n* = 6), uncontrolled intervention development studies (*n* = 8), and one implementation study. Detailed study findings on the effectiveness of DBT are listed by study design in Table 1 and Additional file 1: Appendix 2.

#### DBT interventions: inactive/non-specialist comparators

As shown in Table 1 and Additional file 1: Appendix 2, 13 studies involved comparisons with an inactive or non-specialist treatment control, such as TAU or waitlist. Of these 13 studies, 12 were RCTs and one a non-randomised study with a contemporaneous comparator. Six of these had identified primary outcomes, including self-harm, symptoms, global distress, and hospital admission, and DBT was found to be superior to comparators on some but not all of these outcomes (sample sizes ranging from 20 to 100 participants). Across all 13 studies, there was again a mixture of findings, some suggesting superiority for DBT and some no clear difference (*see* Table 1 and Additional file 1: Appendix 2).

#### DBT interventions: specialist comparators

For studies comparing DBT with other forms of specialist psychotherapy, including General Psychiatric Management, Community Treatment by Experts, Comprehensive Validation Therapy plus 12 step programme, and clinical case management (*n* = 8), DBT was not superior to comparators on the majority of outcomes in RCTs (*n* = 6) and non-randomised studies with contemporaneous comparators (*n* = 2) (*see* Table 1 and Additional file 1: Appendix 2). For studies with specified primary outcomes, DBT showed similar or less improvement in self-harm and suicidality compared to controls in 2/3 RCTs, but was superior to Community Treatment by Experts on suicide attempts in the third RCT. Three of these RCTs had sample sizes greater than 100.

#### DBT interventions: partial or modified comparators

Table 1 and Additional file 1: Appendix 2 include 19 studies that investigated partial or modified DBT therapies. In these studies, DBT was superior to comparators on some outcomes in RCTs (*n* = 6), including three RCTs with sample sizes greater than 100 and one pilot RCT, but inferior to controls on all outcomes in one non-randomised trial with a contemporaneous comparator. No study that investigated partial or modified DBT therapies had both a specified primary outcome and a control group (*n* = 19).

#### DBT interventions in samples not defined only by “personality disorder”

As shown in the Table 1 and Additional file 1: Appendix 2 cohort diagnoses and demographics columns, seven of the studies so far described focused on samples defined by having comorbid conditions in addition to a “personality disorder” diagnosis (severe mental illness (*n* = 1) or substance misuse (*n* = 2)). Four DBT studies used criteria other than “personality disorder” diagnosis, including emotional dysregulation (*n* = 1), parasuicidal behaviours in the past six months (*n* = 1), and severe difficulty in functioning together with frequent suicide attempts (*n* = 1), or crisis service use (*n* = 1). These studies included one RCT, one intervention development study, and five studies involving only pre-post comparisons.

#### DBT intervention: adaptions for specific populations

Additionally, a total of eight studies examined the effectiveness and/or feasibility of DBT adapted for specific clinical or demographic populations, including people with comorbid PTSD (*n* = 3) or eating disorders (*n* = 1), young adults (*n* = 2), female caregivers of children under 3 (*n* = 1), and married men receiving couples therapy *n* = 1). Three of these studies were RCTs. One of the RCTs had 193 participants and found DBT-PTSD to be superior to Cognitive Processing Therapy (CPT) for participants with complex PTSD and a history of childhood abuse, on the primary outcome, PTSD diagnosis, as well as secondary outcomes. In a non-randomised controlled study with 118 participants, DBT was superior to CBT on some primary outcomes and most non-primary outcomes.

#### DBT interventions: summary

Overall, DBT tended to be superior or not different in outcomes from inactive/non-specialist comparators. Findings for specialist DBT and modified DBT treatments were mixed. DBT interventions adapted to specific populations were superior to comparators on most outcomes. Out of the 66 studies investigating the effectiveness of DBT only eight had sample sizes greater than 100 and of these only four were RCTs. Power calculations were rare, limiting interpretation of findings of no difference.

### Cognitive and behavioural and schema therapies

Table 2, Additional file 1: Appendix 3, and Appendix 7 present study characteristics and findings of cognitive and behavioural and schema therapies (*n* = 55). There were 26 RCTs, 17 uncontrolled intervention development studies, three non-randomised studies with contemporaneous controls, and nine uncontrolled studies making only pre-post-treatment comparisons. Detailed findings of the effectiveness of cognitive and behavioural and schema therapies by study design are listed in Table 2 and Additional file 1: Appendix 3 as well as summarised below.

#### Cognitive and behavioural and schema therapy interventions: inactive/non-specialist comparators

Nineteen studies of interventions based on cognitive and behavioural principles and/or the related schema approaches had inactive/non-specialist comparators as shown in Table 2 and Additional file 1: Appendix 3. In RCTs (*n* = 19 including 4 pilot studies), compared to inactive/non-specialist controls, participants receiving cognitive and behavioural or schema therapy showed improvement on some outcomes. 12/19 RCTs had specified primary outcomes, with sample sizes ranging from 34 to 480. Cognitive and behavioural or schema therapy was superior compared to controls on primary outcomes in some studies, including for “personality disorder” symptoms (*n* = 3), “recovery” (*n* = 1), and symptom severity and social functioning in 1/2 RCTs. Cognitive and behavioural or schema therapy was not shown to be superior for other primary outcomes, including depressive or (social) anxiety symptoms (*n* = 1), service use (*n* = 1), and/or self-harm (*n* = 4) (*see* Table 3 and Additional file 1: Appendix 3).

#### Cognitive and behavioural and schema therapy interventions: specialist comparators

In studies with specialist treatment comparators, including Rogerian Supportive Therapy, Transference-Focused Therapy, Dynamic psychotherapy, group-based CBT, individual Cognitive-Evolution Therapy, Mindful Emotion Awareness and Cognitive Reappraisal, and different treatment settings (*n* = 7), cognitive and behavioural therapy was inferior to or showed similar improvements to control treatments for all outcomes in RCTs (*n* = 4) and non-randomised studies with contemporaneous comparators (*n* = 3) (*see* Table 2 and Additional file 1: Appendix 3). This included the results of three RCTs and two non-randomised studies with contemporaneous controls with specified primary outcomes (“BPD” symptoms, symptom severity, personality functioning, and interpersonal problems). Sample sizes of studies with primary outcomes ranged from 46 to 205.

#### Cognitive and behavioural and schema therapy interventions: partial or modified interventions

Table 2 and Additional file 1: Appendix 3 report three RCTs, including one pilot RCT, which examined modifications of cognitive and behavioural or schema therapies. Modifications included addition of phone support, or therapeutic assessments, and interventions delivered at home. These interventions were not superior to unmodified comparators on any outcomes, including the primary outcome in the two RCTs which reported these: one study with 20 participants found no difference in “BPD” recovery with the addition of phone support to schema therapy, and one study with 62 participants found mixed findings on the primary outcome suicidality with delivery of a CBT-based treatment at home.

#### Cognitive and behavioural and schema therapy interventions in samples not defined only by “personality disorder”

As shown in Table 2 and Additional file 1: Appendix 3, of the above studies nine examined the effectiveness of cognitive and behavioural treatments for clinical populations with “personality disorder” diagnoses and comorbid mental health problems, or individuals with related difficulties but not a formal “personality disorder” diagnosis. These studies looked at individuals with “BPD” symptoms and comorbidities including substance use (*n* = 1) and mood disorder (*n* = 4), or at populations that met our criteria for difficulties that appeared comparable to those of people receiving “personality disorder” diagnoses (*n* = 4), including repeated self-harm (*n* = 1), non-suicidal self-injury disorder (*n* = 1), previous suicide attempts (*n* = 1), and history of childhood sexual abuse (*n* = 1).

#### Cognitive and behavioural and schema therapy interventions: summary

Overall, cognitive and behavioural and schema interventions with inactive/non-specialist comparators showed showed improvements on only some measures compared to controls. Studies with active/specialist comparators and studies investigating modified interventions were inferior to or showed similar improvements to controls. Of the 55 studies investigating the effectiveness of cognitive and behavioural and schema treatments, nine studies included > 100 participants of which seven were RCTs (six with inactive/non-specialist comparators).

### Psychodynamic and MBT studies

Table 3, Additional file 1: Appendix 4, and Appendix 8 summarise studies investigating the effectiveness of MBT (*n* = 20) and psychodynamic interventions (*n* = 54). There were 25 RCTs, and 48 non-randomised studies, which included non-randomised studies with contemporaneous controls (*n* = 17) and studies without control groups making only pre-post comparisons (*n* = 31). One uncontrolled study focused on intervention development.

#### MBT interventions: inactive/non-specialist comparators

As shown in Table 3 and Additional file 1: Appendix 4, four RCTs compared MBT with an inactive/non-specialist treatment control (as did a non-randomised study comparing with a historical cohort). MBT was superior to the inactive/non-specialist controls on most outcomes. Two RCTs specified primary outcomes, and MBT proved superior in reducing both “BPD” symptoms (*n* = 1) and suicide attempts (*n* = 1). The 2/4 RCTs with primary outcomes included 41 and 51 participants.

#### MBT interventions: specialist comparators

For studies comparing MBT with other forms of specialist treatment, including specialist TAU, supportive group therapy, Structured Clinical Management, and DBT, (*n* = 9), MBT showed no significant difference in most outcomes in 3/4 RCTs, with the fourth (sample size 107) reporting greater improvements in the primary outcomes of parasuicidal behaviours and number of hospitalisations compared with Structured Clinical Management (*see* Table 3 and Additional file 1: Appendix 4). In three non-randomised studies, results were mixed with few additional benefits reported for MBT compared with other specialist treatments. In primary outcomes, one study reported similar reductions in bed days to the specialist treatment comparator.

#### MBT interventions: treatment setting comparisons

Table 3 and Additional file 1: Appendix 4 include one RCT comparing MBT delivered in different settings (sample size 114) which found no differences on primary (symptom severity) or secondary outcomes between MBT at a day hospital compared to an intensive out-patient MBT.

#### Psychodynamic interventions: inactive/non-specialist comparators

Table 3 and Additional file 1: Appendix 4 show 13 studies on psychodynamic treatments with inactive/non-specialist comparators including six RCTs and seven non-randomised studies. Participants receiving psychodynamic therapy showed greater improvements compared to inactive/non-specialist comparators in the majority of outcomes in RCTs and close to all outcomes in non-randomised studies with control groups. Greater improvement in the primary outcome than control was reported in the 2/3 RCTs (sample sizes 27–62) and all four non-randomised studies (sample sizes 45–143) that specified a primary outcome (*see* Table 3 and Additional file 1: Appendix 4).

#### Psychodynamic interventions: specialist comparators

In studies with specialist comparators (*n* = 11), including manual-based Psychiatric-Psychodynamic sessions, General Psychiatric Management, cognitive therapy, and Transference-Focused Therapy plus supportive treatment, the intervention group was superior to the control group on only a few outcomes in RCTs (*n* = 8), but most outcomes in non-randomised studies (*n* = 3) (*see* Table 3 and Additional file 1: Appendix 4). Of RCTs specifying primary outcomes (sample sizes 25–99), only 1/3 RCTs reported greater progress in therapy (*n* = 1) compared to specialist comparators, and one RCT did not report differences between groups in symptom severity and interpersonal symptoms. One non-randomised study reported greater improvement in “personality disorder” symptoms for Dynamic Deconstructive Psychotherapy compared to controls and DBT.

#### Psychodynamic interventions: treatment setting comparisons

Table 3 and Additional file 1: Appendix 4 list the six non-randomised studies which compared the outcomes of psychodynamic therapy delivered in different settings. There was no difference in outcomes, including the primary outcome symptom severity, in four studies comparing day hospital, outpatient, and inpatient services (sample sizes 143–371). However, community and step-down services were superior to residential services on all outcomes.

#### Psychodynamic and MBT interventions in samples not defined only by “personality disorder”

As shown in Table 3 and Additional file 1: Appendix 4, of the above studies, six focused on clinical populations with “personality disorder” diagnoses and comorbid mental health problems, or individuals with related difficulties but not a formal “personality disorder” diagnosis. Study samples included people with alcohol use and comorbid “personality disorder” diagnosis (*n*-1) with treatment resistant depression and a history of early childhood trauma together with comorbid “personality disorder” diagnosis (*n* = 1) and with poor personal, social, and/or interpersonal functioning with or without “personality disorder” diagnosis (*n* = 4).

#### Psychodynamic and MBT interventions: adaptions for specific populations

Additionally, two RCTs (*n* = 30), one being the follow-up study, examined the effectiveness of psychodynamic treatments that were adapted to specific clinical or demographic populations. Compared to controls, Dynamic Deconstructive Psychotherapy adapted for people with a “BPD” diagnosis and active alcohol use or dependence, was superior on the majority of outcomes, including all primary outcomes (“BPD” symptom severity, parasuicidal behaviour, alcohol misuse, and institutional care).

#### Psychodynamic interventions: summary

Overall, psychodynamic and MBT interventions were superior to inactive/non-specialist comparators on most outcomes. Compared to specialist therapies, MBT and psychodynamic interventions tended to be similar on most outcomes in RCTs. While psychodynamic interventions were superior to specialist comparators on most outcomes in non-randomised studies, there were mixed findings for non-randomised studies investigating the effectiveness of MBT. In studies comparing different treatment settings, there was some evidence of superiority of community and step-down over residential services. There was no difference in the effectiveness between MBT settings. Lastly, psychodynamic interventions adapted to specific populations were superior to comparators on most outcomes. 23/74 studies investigating the effectiveness of psychodynamic and MBT interventions included > 100 participants of which seven were RCTs (three of which had specialist comparators).

### Other studies

Table 4, Additional file 1: Appendix 5, and Appendix 9 present studies on any treatment type other than the psychotherapies listed above (*n* = 41). These included studies of mixed therapeutic modalities (*n* = 10), other individual therapies (*n* = 7), social-interpersonal and functional therapies (*n* = 5), self-management and care planning interventions (*n* = 6), as well as studies investigating outcomes of different approaches to service design and delivery (*n* = 13). Most studies were RCTs (*n* = 25), while three studies made comparisons with contemporaneous control groups, and 13 only pre-post comparisons. Table 4 and Additional file 1: Appendix 5 list detailed findings of studies on the effectiveness of other interventions by study design. Findings are summarised below.

#### Mixed interventions

As shown in Table 4 and Additional file 1: Appendix 5, in RCTs with inactive/non-specialist comparators examining mixed therapeutic modalities, the intervention group was superior to controls on most outcomes (*n* = 3), including the primary outcomes (drop out and suicide attempts) of an RCT with 104 participants, but not “BPD” symptoms, the primary outcome of an RCT with 71 participants.

In one RCT with a specialist comparator, cost-effectiveness did not differ between the step-down treatment and outpatient control group (0/1).

#### Other individual therapies

Compared to controls, participants receiving individual therapies other than the psychotherapies listed above (including Art therapy, Abandonment psychotherapy, Body Awareness Group therapy, short-term psychotherapy, and psychoeducation) showed greater improvements in close to all outcomes in RCTs with inactive/non-specialist comparators (*n* = 5 including one pilot RCT). However, in the two RCTs with specified primary outcomes Abandonment psychotherapy was superior to the control for suicidal relapse and hospitalisation (*n* = 1), but psychoeducation was not superior to control for “BPD” severity (*n* = 1).

Other individual therapies were not superior to controls in two RCTs with specialist treatment comparators, including one RCT included one RCT comparing to Abandonment psychotherapy delivered by nurses instead of trained psychotherapists, and another comparing to Transference-Focused Therapy and DBT, on all outcomes including primary outcomes.

Sample sizes of RCTs with primary outcomes ranged from 50 to 170.

#### Social-interpersonal and functional interventions

Table 4 and Additional file 1: Appendix 5 show that similar results were found for social and interpersonal interventions, with the intervention group being superior compared to controls on up to half of the outcomes in RCTs with inactive/non-specialist comparators (*n* = 3). Additionally, the intervention group was superior on primary outcomes in only 1/2 RCTs with identified primary outcomes: Psychoeducation plus problem-solving therapy showed greater improvement in social functioning and social problem-solving skills compared to waitlist, however, the cognitive rehabilitation and psychoeducation groups improved similarly in general functioning. RCTs with primary outcomes included 70 and 176 participants.

There were no between-group differences found in RCTs with specialist comparators, including delayed Functional Imagery Training and Social Skills Training in the clinic/hospital only (*n* = 2 including one pilot RCT).

#### Self-management and care planning

There were no between-group differences on outcomes in 1/2 RCTs on self-management and care planning compared to self-management only or established generic or specialist mental health services (*n* = 1). As shown in Table 4 and Additional file 1: Appendix 5, this included the primary outcomes of two RCTs: The Joint Crisis Plan group and TAU group had similar rates of self-harm in one RCT including 88 participants (the RCT was not powered to find an effect). The second RCT with a sample size of 52 found no difference in service admissions in Nidotherapy-enhanced assertive outreach compared to standard assertive outreach. In one RCT without identified primary outcomes, compared to Structured Goal-Focused Pre-Treatment Intervention (GFPTI), participants receiving therapeutic assessment improved in more than half of the outcomes.

#### Novel mental health service models compared to day hospital

Regarding service design models, one RCT comprising four papers comparing step-down treatment with outpatient treatment showed no between-group differences on outcomes, including a range of primary outcomes (*see* Table 4 and Additional file 1: Appendix 5). In the other RCT only including patients with a “BPD” diagnosis, the step-down intervention group was superior compared to the outpatient group on half of the outcomes.

Lastly, two RCTs with samples > 100 examining novel mental health service models compared to established generic or specialist mental health services found the intervention group to be superior on some outcomes compared to the control group, but on primary outcomes related to service use only in 1/2 RCTs.

#### Other treatments in samples not defined only by “personality disorder”

As depicted in the Table 4 and Additional file 1: Appendix 5 cohort columns, six of the above studies on other treatments focused on specific populations, including three RCTs, one non-randomised study with a contemporaneous control, and one uncontrolled study making only pre-post comparisons. One RCT compared the effectiveness of Abandonment psychotherapy and intensive TAU for individuals with major depression and a comorbid “BPD” diagnosis. Another RCT investigated a joint crisis plan and TAU for young people without a “personality disorder” diagnosis but at least two episodes of self-harm in the previous three months. A third RCT compared the effectiveness of Nidotherapy and TAU for individuals with severe mental illness and a comorbid “personality disorder” diagnosis. One non-randomised study examined collaborative care management and TAU for individuals with major depression with or without a comorbid “personality disorder” diagnosis. Lastly, an uncontrolled study investigated emotion regulation skills training for a community-based sample of individuals with adverse childhood experiences over time.

## Discussion

Our scoping review collated quantitative evidence regarding community-based psychological, psychosocial, and service level interventions designed for people with CEN. Most studies focused on people given a “personality disorder” diagnosis, with a small number relating to people who appeared to have comparable difficulties (6%). Some observations may be made from this literature, but large gaps are prominent.

### What *does* the literature tell us?

We identified 226 papers reporting on 210 distinct studies carried out in a range of countries, the majority in Europe or North America. The largest group of studies evaluated the effectiveness of DBT, followed by psychodynamic therapy, cognitive and behavioural therapy, MBT, and schema therapy. Research on psychological treatments dominated, with only a small handful of studies using any method to investigate interventions with primarily social targets, self-management, care planning, or models of service delivery.

The total quantity of studies, given the breadth of the search and inclusion of uncontrolled studies and studies with very small samples, is small. Little literature was published in the twentieth century, with most included studies published after 2005, since when annual publication rates have slowly risen. This may reflect a shift internationally away from the view of “personality disorder” as untreatable and justifying exclusion from mental health services that prevailed in the twentieth century [259]. In the early 2000s, factors including the publication of trials that held out prospects for successful treatment, service user activism, and key policy documents such as the UK’s “Personality Disorder: No longer a diagnosis of exclusion” may have contributed to greater confidence that research in this area is potentially fruitful [6, 260, 261]. However, stigma, therapeutic pessimism, and difficulty accessing any kind of helpful care are still widely reported [10, 11, 14, 20, 262]. The results of our searches suggest that investment in large well-designed studies that test clear primary hypotheses has remained very limited around the world, which may reflect a continuing lack of optimism, and the impacts of the particularly severe stigma that appears associated with CEN.

The evidence base that has been established thus far relates mainly to specialist psychotherapies, delivered especially to people with a “BPD” diagnosis. Many studies are small and/or non-randomised, but studies with any methodology have tended to suggest benefits for specialist psychotherapies of a range of types compared with inactive/non-specialist controls, both in studies focused on people with a “BPD” diagnosis and with broader groups. However, results do not tend to suggest one kind of specialist treatment is clearly superior to another – this coheres with the results of more narrowly focused systematic reviews that do not identify a clear gold standard but suggest a variety of psychological treatments are helpful for those who engage with them [23, 26]: a focus on what works well for whom, and why, would be helpful in further work.

Contrary to the pessimistic outlook often reported regarding potential for improvement among people with a “personality disorder” diagnosis, a large majority of studies involving before and after comparisons find significant reductions in symptoms and self-harm as well as improvements in other outcomes. This seems to be the case across treatment types as well as diagnoses, often to the extent that a substantial minority of participants were assessed as no longer meeting criteria for a “personality disorder” diagnosis. Study methods often made it hard to assess how far this was a result of treatments received, including those being investigated, and how far of the natural improvement in symptoms and difficulties (people may also tend to be recruited to studies at times when difficulties are especially severe). Findings from these studies suggest the value of uncontrolled studies and of before and after treatment comparisons is very limited except where the main purpose is to test the feasibility and acceptability of delivering an intervention: it appears likely that improvement will be found whatever interventions are offered.

Regarding specific populations such as those who are younger or older or who have some of the conditions that are frequently comorbid with CEN, such as substance misuse or psychosis, we found substantial numbers of interesting small studies, mainly aimed at intervention development, or establishing that treatments are feasible and acceptable in specific populations. These provide potential building blocks for further design and testing of interventions in important populations where substantial trials have yet to be reported.

### What does the literature *not* tell us?

Gaps in the evidence needed to underpin high quality service delivery for people with CEN are large. Service users and clinicians report that mental health care systems appear ill-equipped to deliver accessible care of high quality [10, 15, 20], yet there are hardly any published investigations of the best approaches to designing teams and systems. Care planning, crisis planning, and self-management are to a large extent not investigated as applied to people with CEN. We identified very few studies of interventions with social targets, including employment and social relationships, even though people with CEN identify these as a priority [14, 263]. We found very little evidence of co-production or service user leadership in either research or intervention design, despite the benefits of these in producing research that aligns to service user needs and priorities [264]. We also found very little quantitative research on either trauma-informed care for this group, or interventions for people with comorbid PTSD, despite calls to place trauma at the centre of thinking about CEN [14, 262, 265].

Only a few studies evaluated treatments adapted to specific populations of interest, such as younger or older age groups, parents or patients with comorbid severe mental illnesses, substance misuse, or childhood trauma. As above, a number of small-scale initial studies appeared promising, but were limited by small sample sizes and/or observational or feasibility/intervention development study designs. Lack of more substantial evaluations of well-designed interventions for these groups who have tended to be still more under-served than others with CEN appears an important gap.

Most studies were conducted with participants with a “BPD” diagnosis, so that there is little evidence on effective interventions for people with other diagnoses, or who may have comparable difficulties but not have received a diagnosis. Samples are largely White and female with close to no papers focusing on diverse gender and sexual identities (despite some evidence of LGBTQ + groups being more likely to receive a “personality disorder” diagnosis [266, 267]), or other ethnicities. Studies generally measured effectiveness of interventions by examining improvement in whether diagnostic criteria continued to be met for “personality disorder”, symptom outcomes, self-harm, and service use. However, outcomes prioritised by service users such as personal achievements, employment, and social connections were reported much less [263], and the possibility of iatrogenic harm was also rarely examined. Interventions addressing social needs are especially important in the light of findings of longitudinal studies showing that while symptoms and suicidal behaviour tend to improve with time, this is less the case for psychosocial functioning including rate of employment [268, 269]. Implementation studies examining how to embed successful interventions in real-world settings were also largely absent.

### Limitations

Despite the breath of our approach, the findings of the present review must be considered in light of several limitations. In order to provide an overview of evidence acquired so far and identify gaps, we have created broad, often heterogenous, categories of study designs. This approach is inevitably superficial and limits how far meaningful comparisons can be made across study types, treatments, and subpopulations. In keeping with scoping review methodology recommendations, we did not formally assess the quality of the studies, although we have commented on some obvious limitations, for example relating to small trial populations or uncontrolled study designs.

Additionally, while inclusion criteria were kept broad, and a variety of search terms applied to try to include studies with paalthough the number of studies excluded on these grounds was smallrticipants with any diagnosis of “personality disorder” as well as those with comparable difficulties, capturing the latter reliably is likely to have been particularly difficult, and only a small number of studies not based on such criteria were included. We also have not included many studies that are transdiagnostic or include mixed populations of mental health service users. While our search strategy was developed by a team of researchers, clinicians, people with relevant lived experience, and an information scientist, it was not peer reviewed. Lastly, in order to make this very broad search feasible, we included only studies published in English as well as published and peer-reviewed evidence, excluding pre-prints and theses. This may well have excluded some relevant evidence, although the number of studies excluded on these grounds was small.

## Conclusions

Our overall conclusion from this scoping review is that people with CEN, despite being numerous among community mental health service users [270] have thus far been poorly served by clinical research. Mental health research is in general under-funded compared with other areas of health [271]. Our findings suggest that this is especially striking in the field of CEN, in which little was published prior to 2005 and the tally has increased only gradually subsequently, now only just exceeding two hundred quantitative studies including 96 RCTs of community and outpatient interventions, even including studies of any scale using any method.

Much therefore needs to be done to develop a robust evidence base in this area, especially beyond a narrow focus on specialist psychotherapies for people with a “BPD” diagnosis, where a substantial number of trials have resulted in a finding that several specialist therapies appear better than treatment as usual, but not in a clear finding that any treatment is clearly superior. Future research should address outcomes valued by patients rather than being limited to a focus on self-harm and symptoms: relevance to service users is much more likely to be achieved by the adoption of co-production in design of both interventions and research studies. The recent service user-led StopSIM campaign against the Serenity Integrated Monitoring intervention [272], which involved the police in responses to some people with frequent contact with emergency services, exemplifies the potential for iatrogenic harm from interventions that are unevaluated, or where the potential for harm has not been assessed. Research on important populations such as older and younger people and people with major comorbidities, and on interventions focusing on people with CEN as parents, partners or relatives needs to progress beyond the feasibility studies conducted so far. Larger and more diverse samples are needed to be confident of relevance across service user populations.

Models of service delivery have been largely neglected in research so far despite recurrent complaints from service users and clinicians that current systems are fragmented and inaccessible. Realist evaluations may shed a light on what mechanisms underly the effectiveness of different interventions as well as what type of intervention works for which patient group and in what context. Relevant contexts may be individual, such as personal life and stage of life, as well as systemic. Additionally, services need to deliver holistic and person-centred care that addresses service users’ interconnected needs and intersecting experiences over several years: large-scale observational designs may be helpful in understanding outcomes over longer periods [10, 14]. Lastly, patients and carers with relevant experiences need to be invited to co-produce the development and evaluation of treatments to not only ask the right questions but also examine these in a meaningful way (Table 5).

**Table 5:** Lived experience commentary written by Sarah Labovitch and Jennie Parker

In light of the Community Mental Health Framework (CMHFA), this review is well timed to revise thinking around what *should* be available to people who may meet the diagnostic criteria for “personality disorder”/CEN. It may also prompt researchers and service-providers to consider what is important to us—it was disappointing to see that only 44/226 studies reported on quality of life, whilst most primary outcomes focused on diagnostic-related criteria
Time to follow-up in many studies discussed is limited. Side-effects of funding constraints typically lead to quantitative research and RCTs being prioritised. We agree with the question of what underlies reported improvements, and would say this is not just in relation to observational studies. It would be interesting to delve further into this
Despite advancements in recent years, community service-provision for “personality disorder”/CEN is nevertheless lagging behind other areas of mental health. Treatment in the community must be patient-centred: adapted to factors such as age, culture, comorbidity, substance misuse and trauma. Some health professionals still display discriminatory attitudes towards CEN, or simply don’t know how to help. Finding a clinician with the right skills and compassion is depressingly arduous. Further, exclusion criteria and high thresholds can make “specialist” services inaccessible. Meanwhile, the notion of individuals actually having a choice in therapist is vanishingly slim, adding to the risk of iatrogenic harm and a “cliff-edge” of care. Services need to commit to consistent long-term contact, as well as tailoring treatment to individual needs
As with others, we have experienced stigma, rejection, and repeated/inappropriate referrals. This paper leaves us with a conundrum, both in relation to the integrated approach proposed by the
CMHFA and access to good and timely support. Whilst this is a scoping review of quantitative research, our recommendation is for further investigation into the active ingredients of therapy: what makes good outcomes for some but not others, the importance of the relationship, and whether we have a choice of therapist (considerate of age, culture, gender, etc.) or of intervention. We also noted the limited research on peer support, compared to our experience of its value. With such a diverse population and diverse range of therapies (and variance within specific models), clearer guidance would be helpful so that we can all make fully-informed choices

## Supplementary Information

**Additional file 1: Appendix 1.** Search strategy. **Appendix 2.** Dialectical Behavioural Therapy (DBT) treatments. **Appendix 3.** Cognitive and Behavioural Therapy and Schema Therapy treatments. **Appendix 4.** MBT and Psychodynamic Therapy treatments. **Appendix 5.** Other treatments. **Appendix 6.** Table of studies testing Dialectical Behavioural Therapy (DBT) treatments. **Appendix 7.** Table of studies testing Cognitive and Behavioural Therapy and Schema Therapy treatments. **Appendix 8.** Table of studies testing MBT and Psychodynamic Therapy treatments. **Appendix 9.** Table of studies testing other treatments.

## Data Availability

All data generated or analysed during this study are included in this published article [and its supplementary information files].
